# Comparative Electrochemical Noise Characterization of Ti-CP2, Ti-6Al-2Sn-4Zr-2Mo, and Ti-6Al-4V in Ringer’s Solution Using Statistical and Hilbert–Huang Analyses

**DOI:** 10.3390/ma19204337

**Published:** 2026-10-15

**Authors:** Citlalli Gaona-Tiburcio, Erick Maldonado-Bandala, Jesús Manuel Jáquez-Muñoz, Demetrio Nieves-Mendoza, Ce Tochtli Méndez-Ramírez, Bruno Almeraya-Gaona, Laura Landa-Ruiz, Brenda Baltazar-Garcia, Francisco Estupinan-Lopez, Luis Daimir Lopez-Leon, Javier Olguin-Coca, Facundo Almeraya-Calderón

**Affiliations:** 1 Centro de Investigación e Innovación en Ingeniería Aeronáutica (CIIIA)/Facultad de Medicina, Facultad de Ingenieria Mecanica y Eléctrica, Universidad Autónoma de Nuevo León, San Nicolás de los Garza 66455, Mexico; bruno.almerayag@uanl.edu.mx (B.A.-G.); francisco.estupinanlp@uanl.edu.mx (F.E.-L.); facundo.almerayacld@uanl.edu.mx (F.A.-C.); 2 Facultad de Ingeniería Civil, Universidad Veracruzana, Xalapa 91000, Mexico; erimaldonado@uv.mx (E.M.-B.); dnieves@uv.mx (D.N.-M.); lalanda@uv.mx (L.L.-R.); pao.baltazar.08@gmail.com (B.B.-G.); 3 Instituto Tecnológico de Ciudad Juárez, Metal-Mechanics Department, Av. Tecnológico, 1340, Ciudad Juárez 32500, Mexico; jesus.jm01@cdjuarez.tecnm.mx; 4 Área Académica de Ingeniería y Arquitectura, Universidad Autónoma del Estado de Hidalgo, Carretera Pachuca-Tulancingo Km. 4.5, Hidalgo 42082, Mexico; luis_lopez@uaeh.edu.mx (L.D.L.-L.); olguinc@uaeh.edu.mx (J.O.-C.)

**Keywords:** titanium alloys, Ti-CP2, electrochemical noise, Hilbert–Huang transform, empirical mode decomposition, Ringer’s solution, SEM/EDS

## Abstract

Titanium and its alloys are widely used in biomedical applications because of their favorable mechanical properties, biocompatibility, and corrosion resistance. This study comparatively evaluated the electrochemical behavior of Ti-CP2, Ti-6Al-2Sn-4Zr-2Mo, and Ti-6Al-4V in Ringer’s solution using electrochemical noise (EN), empirical mode decomposition (EMD), Hilbert–Huang Transform (HHT), and scanning electron microscope/Energy-dispersive X-ray spectroscopy (EDS) SEM/EDS characterization. Statistical EN analysis revealed marked differences among the investigated materials. Ti-6Al-2Sn-4Zr-2Mo exhibited the highest noise resistance (45,412 ± 57 Ω·cm^2^), followed by Ti-6Al-4V (11,784 ± 39 Ω·cm^2^) and Ti-CP2 (4114 ± 45 Ω·cm^2^), whereas relatively low localization index values were obtained for all three materials (0.02634–0.06). EMD revealed four, five, and six intrinsic mode functions for Ti-CP2, Ti-6Al-2Sn-4Zr-2Mo, and Ti-6Al-4V, respectively. Hilbert time–frequency–amplitude analysis further demonstrated distinct temporal and frequency distributions of the electrochemical fluctuations, with Ti-6Al-4V exhibiting the broadest and most persistent distribution of transient activity. SEM/EDS revealed localized post-exposure compositional heterogeneity and different degrees of accumulation of electrolyte-derived species but was not interpreted as direct evidence of passive-film chemistry or integrity. Overall, the combined statistical EN, EMD, Hilbert time–frequency, and SEM/EDS results demonstrate distinct passive electrochemical responses among the three titanium materials, with Ti-6Al-2Sn-4Zr-2Mo exhibiting the highest resistance to electrochemical fluctuations under the investigated conditions.

## 1. Introduction

Titanium alloys are among the most widely used metallic biomaterials because of their excellent combination of mechanical properties, corrosion resistance, and biocompatibility, making them suitable for orthopedic and dental implants as well as other biomedical devices [1]. Depending on their phase constitution, titanium alloys are commonly classified as α, near−α, α + β, and metastable β alloys, each exhibiting distinct mechanical and electrochemical characteristics. Commercially pure titanium (α phase, Ti CP) exhibits excellent corrosion resistance; however, its relatively low mechanical strength limits its application in load-bearing implants. Consequently, alloying elements such as molybdenum, vanadium, niobium, and chromium have been incorporated as β-stabilizers to improve both mechanical performance and corrosion resistance [2]. Among these elements, molybdenum has been reported to significantly enhance corrosion resistance [2]. Zirconium, considered a neutral alloying element, has also attracted considerable interest because it promotes the formation of a more homogeneous and protective oxide layer while reducing material degradation [3]. As a result, near-α titanium alloys such as Ti-6Al-2Sn-4Zr-2Mo exhibit an attractive combination of high mechanical strength, excellent corrosion resistance, and good biocompatibility, making them promising candidates for biomedical applications [4,5].

Recent developments have focused on the design of α + β titanium alloys containing optimized concentrations of β-stabilizing elements to improve mechanical performance while maintaining excellent corrosion resistance and biocompatibility. These alloys generally contain 4–8 wt.% β-stabilizing elements, resulting in an improved balance between strength, ductility, and corrosion resistance [6]. Among them, Ti-6Al-4V remains the most widely used titanium alloy for biomedical applications because of its excellent combination of mechanical properties, corrosion resistance, and long-term clinical performance. Nevertheless, concerns have been raised regarding the potential biological effects associated with the release of aluminum and vanadium ions following long-term exposure or wear under physiological conditions [7,8].

Consequently, considerable research has focused on developing titanium alloys containing alternative alloying elements such as zirconium, tantalum, molybdenum, and tin to further improve corrosion resistance and biocompatibility while reducing the dependence on vanadium-containing systems. Zirconium is particularly attractive because it belongs to the same group as titanium, exhibits excellent corrosion resistance, and is highly biocompatible, making it suitable for orthopedic and dental applications [9,10,11]. In this context, near-α titanium alloys such as Ti-6Al-2Sn-4Zr-2Mo have attracted increasing attention because they combine high mechanical strength with excellent corrosion resistance and have been proposed as promising candidates for biomedical applications.

Although titanium alloys exhibit excellent corrosion resistance, they remain susceptible to electrochemical degradation under aggressive physiological environments containing chloride ions. Physiological electrolytes may locally modify the electrochemical behavior of the protective oxide layer, promoting transient localized corrosion events, while the presence of other dissolved ions influences the overall electrochemical response of titanium alloys [12,13,14,15,16].

Electrochemical techniques are widely employed to investigate the corrosion behavior of titanium alloys in physiological environments. Among them, electrochemical noise (EN) is a non-intrusive technique that records spontaneous potential and current fluctuations generated at the material/electrolyte interface without imposing an external electrochemical perturbation [17,18,19]. The direct analysis of EN time series provides information on the amplitude, occurrence, and temporal distribution of electrochemical transients. In addition, statistical parameters evaluated in the time domain, such as noise resistance (Rn), localization index (LI), skewness, and kurtosis, allow quantitative differences in the electrochemical fluctuations to be identified. However, because corrosion-related transients are inherently non-stationary, their characteristics may change throughout the exposure period, making time–frequency analysis particularly useful for their interpretation.

The Hilbert–Huang Transform (HHT) provides a suitable approach for analyzing non-linear and non-stationary signals such as electrochemical noise. Through the representation of instantaneous frequency as a function of time, HHT enables transient electrochemical events to be resolved in the time–frequency domain and provides information that cannot be obtained from time-domain statistical parameters alone. In passive materials such as titanium alloys, this approach is particularly relevant because spontaneous electrochemical fluctuations may occur over different characteristic time scales as the passive interface undergoes dynamic changes. Therefore, combining EN time-series observations, statistical analysis in the time domain, and HHT analysis in the time–frequency domain provides complementary information on the dynamics of the alloy/electrolyte interface.

Previous studies have demonstrated that the electrochemical behavior of Ti CP2, Ti-6Al-2Sn-4Zr-2Mo, and Ti-6Al-4V in physiological environments is strongly influenced by alloy composition, microstructure, surface condition, and passive-film stability [20,21,22,23,24,25]. Earlier investigations comparing these titanium materials in Ringer’s solution have reported differences in their corrosion response using conventional electrochemical and signal processing approaches [20,21,22]. More recent studies have further demonstrated that microstructural condition, α/β phase distribution, and surface preparation can significantly modify the electrochemical response and passive-film characteristics of titanium alloys [24,25]. These observations emphasize that the electrochemical behavior of passive titanium materials involves dynamic interfacial processes that may not be completely described by conventional electrochemical parameters or by a single representation of the electrochemical signal.

Despite these advances, comparatively less attention has been given to the direct correlation between the temporal characteristics of spontaneous electrochemical-noise transients, their statistical distribution in the time domain, and their evolution in the time–frequency domain for passive titanium materials exposed under identical physiological conditions. Statistical EN parameters provide quantitative information on signal magnitude, asymmetry, and the occurrence of extreme transient events, but they do not identify when these events occur or how their characteristic frequencies evolve during exposure. Conversely, HHT analysis can resolve the temporal evolution of the frequency components associated with these non-stationary fluctuations. Correlating both approaches may therefore provide a more complete description of the dynamic electrochemical response of passive titanium surfaces than either analysis considered independently.

Therefore, the aim of this work was to comparatively characterize the spontaneous electrochemical behavior of Ti-CP2, Ti-6Al-2Sn-4Zr-2Mo, and Ti-6Al-4V exposed to Ringer’s solution under identical experimental conditions using electrochemical noise (EN). Ti-CP2 was included as a reference material to provide a baseline for comparison with the two titanium alloys. The electrochemical response was evaluated through potential and current noise time series, statistical analysis in the time domain, empirical mode decomposition (EMD), and time–frequency analysis using the Hilbert–Huang Transform (HHT). This combined approach allows differences in the amplitude, occurrence, statistical distribution, and temporal evolution of spontaneous electrochemical fluctuations to be identified, while EMD and HHT provide information on the characteristic time scales and the temporal evolution of the frequency content of these events.

The contribution of this study lies in integrating the direct observation of electrochemical noise transients with their statistical characterization in the time domain, their decomposition into characteristic time scales by EMD, and their time–frequency characterization by HHT. Rather than assigning a corrosion mechanism from a single parameter, the combined analysis is used to distinguish differences in the intensity, distribution, and temporal evolution of electrochemical events among Ti-CP2, Ti-6Al-2Sn-4Zr-2Mo, and Ti-6Al-4V in Ringer’s solution. This methodology provides a more comprehensive description of the dynamic electrochemical behavior of the passive interface and allows transient activity potentially associated with local passive-film destabilization and subsequent recovery to be evaluated.

## 2. Materials and Methods

### 2.1. Materials

Commercially pure titanium Grade 2 (Ti-CP2) and Ti-6Al-2Sn-4Zr-2Mo (Ti-6242) supplied by Titanium Engineers (Stafford, TX, USA), and Ti-6Al-4V (ASTM B348-13 Grade 5, AMS 4928W) supplied by Performance Titanium Group (PTG, San Diego, CA, USA), were used in this investigation. The three titanium materials were evaluated in the as-received condition without additional heat treatment or thermomechanical processing. Their chemical compositions were determined by X-ray fluorescence (XRF) spectroscopy using an Olympus DELTA XRF analyzer (Olympus Corporation, Richmond, TX, USA) and are presented in Table 1.

The specimens used for electrochemical testing were prepared using conventional metallographic procedures based on ASTM E3 [26]. The exposed surfaces were sequentially ground using silicon carbide (SiC) abrasive papers from 400 to 800 grit (Struers, Ballerup, Denmark) using Buehler metallographic preparation equipment (Buehler, Lake Bluff, IL, USA). Subsequently, the specimens were ultrasonically cleaned in ethanol for 10 min, rinsed with deionized water, and dried prior to electrochemical testing. The specimens were obtained directly from the commercial titanium materials and were not prepared by powder compaction or pellet pressing.

For microstructural characterization, metallographic specimens were prepared according to ASTM E3 [26]. The specimen surfaces were sequentially ground using SiC abrasive papers up to 4000 grit, followed by ultrasonic cleaning in ethanol and rinsing with deionized water. The polished specimens were subsequently etched with Kroll’s reagent (3 mL HF, 5 mL HNO_3_, and 100 mL H_2_O) according to ASTM E407 [27] to reveal the microstructure.

### 2.2. Microstructural Characterization

Microstructural characterization was performed using an Olympus GX53 optical microscope (Olympus Corporation, Tokyo, Japan) and a JEOL JSM-6510LV scanning electron microscope (JEOL Ltd., Tokyo, Japan). Backscattered electron (BSE) images were acquired at magnifications of 500× and 1000× using an accelerating voltage of 20 kV and a working distance of 14 mm. Energy-dispersive X-ray spectroscopy (EDS) was employed to characterize the local elemental composition of selected microstructural features using an Oxford Instruments X-Max 80 detector (Oxford Instruments, Abingdon, UK).

### 2.3. Electrochemical Techniques

All electrochemical measurements were conducted in Ringer’s solution containing 9.0 g/L NaCl, 0.425 g/L KCl, 0.119 g/L CaCl_2_, and 0.1 g/L NaHCO_3_, prepared using deionized water. The experiments were performed at room temperature (25 ± 2 °C).

Electrochemical noise (EN) measurements were performed in accordance with ASTM G199-09 [28]. Two nominally identical specimens of each material were used as working electrodes (WE1 and WE2), while a saturated calomel electrode (SCE) served as the reference electrode. Electrochemical current noise (ECN) was recorded as the galvanic current flowing between the two working electrodes, whereas electrochemical potential noise (EPN) was measured between one working electrode and the reference electrode. Current and potential fluctuations were simultaneously recorded throughout each experiment [29,30].

Each measurement consisted of 4096 data points acquired at a sampling frequency of 1 Hz. The recorded EN signals were processed using MATLAB R2018a (MathWorks Inc., Natick, MA, USA). Prior to statistical and time–frequency analyses, the DC trend was removed from the original signals using a ninth-order polynomial detrending method, as described in Section 2.3.1. Statistical EN parameters were subsequently calculated as described in Section 2.3.2, whereas the ECN signals were further analyzed by empirical mode decomposition (EMD) and the Hilbert–Huang Transform (HHT), as described in Section 2.3.3.

All EN measurements were carried out using a Gill AC potentiostat/galvanostat/zero resistance ammeter (ZRA) (ACM Instruments, Cumnor, Oxford, UK). Each experiment was performed in triplicate (n = 3), and the results are reported as the mean ± standard deviation.

#### 2.3.1. Polynomial Method

Removal of the direct current (DC) component is an essential preprocessing step in electrochemical noise analysis because baseline drift may affect the statistical characteristics and time–frequency representation of the recorded signals. In the present work, baseline drift was removed by fitting the experimental signal with a ninth-order polynomial and subsequently subtracting the fitted curve from the original signal. The ninth-order polynomial (*p* = 9) was selected following previously reported electrochemical-noise methodologies in which lower polynomial degrees (*p* < 9) were found to leave an appreciable residual DC trend [31]. Although increasing the polynomial order improves trend removal, excessively high orders may also remove useful components of the electrochemical-noise signal; therefore, the polynomial order must provide a balance between sufficient DC trend removal and preservation of the relevant electrochemical fluctuations [32]. The same detrending procedure was applied consistently to all EN records. The detrending procedure is expressed by Equation (1).

(1) $$y_{n}=x_{n}-\sum_{i=0}^{p_{o}}a_{i}n^{i}$$

where $x_{n}$ is the original electrochemical noise (EN) signal, $p_{n}$ is the fitted ninth-order polynomial representing the baseline trend, n is the discrete sample index, and $Y_{n}$ is the detrended electrochemical noise signal used for subsequent analyses [33].

#### 2.3.2. Statistical Analysis

Statistical analysis of the electrochemical noise signals was performed in the time domain to quantitatively characterize the magnitude and distribution of the spontaneous potential and current fluctuations [34]. Noise resistance (Rn) was calculated from the standard deviations of the electrochemical potential noise (EPN) and electrochemical current noise (ECN) signals. The standard deviation, σ, was calculated according to Equation (2):

(2) $$\sigma_{x}=\sqrt{\bar{x^{2}}}=\sqrt{\frac{\sum_{1}^{N}{\left(x_{i}-\bar{x}\right)}^{2}}{N}}$$

where $x_{i}$ represents each value of the time series, $\bar{x}$ is the arithmetic mean, and N is the number of data points. The noise resistance was subsequently calculated according to Equation (3):

(3) $$R_{n}=\frac{\sigma_{v}}{\sigma_{I}}*A$$

where $\sigma_{V}$ and $\sigma_{I}$ are the standard deviations of the potential and current noise signals, respectively. Rn provides a measure of the relationship between spontaneous potential and current fluctuations and has frequently been used as an electrochemical-noise parameter related to the resistance of the corrosion system [34]. Higher Rn values are generally associated with lower electrochemical activity; however, its interpretation should be made together with the characteristics of the EN signals and other statistical parameters.

The localization index (LI) was calculated from the relationship between the standard deviation and the root mean square (RMS) of the electrochemical current noise signal according to Equation (4):

(4) $$LI=\frac{\sigma_{I}}{I_{RMS}}$$

where *I*_RMS_ is the root mean square (RMS) value of the electrochemical current noise and σI is its standard deviation. Traditionally, LI values approaching zero have been associated with uniform corrosion, values between 0.01 and 0.1 with mixed corrosion, and values between 0.1 and 1 with localized or pitting corrosion [35,36,37]. However, these ranges should be considered interpretative guidelines rather than definitive criteria for identifying a corrosion mechanism. Accordingly, in the present study, LI was interpreted together with the other statistical EN parameters and the time–frequency characteristics of the electrochemical noise signals.

Skewness and kurtosis were calculated to characterize the statistical distribution of the electrochemical current noise (ECN) fluctuations. Skewness, corresponding to the third standardized statistical moment, describes the asymmetry of the signal distribution, whereas kurtosis, related to the fourth standardized moment, describes the contribution of extreme values and the occurrence of intermittent high-amplitude events. These parameters were calculated according to Equations (5) and (6), respectively [38,39]:

(5) $$skewness=\frac{1}{N}\sum_{i=1}^{N}\frac{{{(x}_{i}-\bar{x})}^{3}}{\sigma^{3}}$$

(6) $$kurtosis=\frac{1}{N}\sum_{i=1}^{N}\frac{{{(x}_{i}-\bar{x})}^{4}}{\sigma^{4}}$$

Previous studies have associated characteristic skewness and kurtosis values with different corrosion processes [38,39]. Nevertheless, these statistical parameters are sensitive to the distribution and magnitude of electrochemical transients and should not be regarded as unequivocal indicators of a specific corrosion mechanism. Accordingly, in the present study they were primarily used to compare the statistical characteristics of the EN signals and were interpreted together with the time-series and HHT results.

The statistical parameters are subject to uncertainty associated with the finite number of data points. The standard error (SE) was estimated according to Equation (7), where N represents the number of data points considered in the analysis [40]:

(7) $$SE=\sqrt{\frac{24}{N}}$$

For the number of data points used in the present study, the calculated SE was 0.077. This value was considered as an estimate of the statistical uncertainty associated with the analyzed EN data.

#### 2.3.3. Hilbert–Huang Transform (HHT)

The Hilbert–Huang Transform (HHT) was applied to further characterize the non-stationary electrochemical noise signals in the time–frequency domain. Unlike conventional frequency-domain approaches, HHT enables the instantaneous frequency content of transient events to be evaluated as a function of time. The method combines Empirical Mode Decomposition (EMD) with the Hilbert transform and is particularly suitable for non-linear and non-stationary signals [33,41].

In the first stage, EMD decomposes the detrended ECN time series into a finite number of intrinsic mode functions (IMFs) and a residual component according to Equation (8):

(8) $$x\left(t\right)=\sum_{i=1}^{N}h^{\left(i\right)}\left(t\right)+d(t)$$

where $h_{i}\left(t\right)$ represents the i-th IMF and $d\left(t\right)$ is the final residual or trend component. Each IMF must satisfy two conditions: (i) the number of extrema and zero crossings must either be equal or differ by no more than one, and (ii) the mean value of the envelopes defined by the local maxima and minima must be approximately zero [42,43]. The decomposition separates oscillatory components occurring at different characteristic time scales without requiring a predefined basis function.

Subsequently, the Hilbert transform is applied to each IMF to obtain its instantaneous characteristics, as expressed in Equation (9):

(9) $$y_{j}\left(t\right)=\frac{1}{\pi }p\int_{-\infty }^{\infty }\frac{h_{j}(\tau )}{t-\tau }d\tau$$

where $y_{j}\left(t\right)$ is the Hilbert transform of the corresponding IMF, $h_{j}\left(t\right)$, and the integral is evaluated in the Cauchy principal-value sense. From the original IMF and its Hilbert transform, an analytical signal can be constructed to determine the instantaneous amplitude and instantaneous frequency as functions of time. The resulting Hilbert spectrum therefore provides a time–frequency representation of the electrochemical noise signal, allowing transient electrochemical events and their characteristic frequencies to be localized during the exposure period [44,45,46,47].

## 3. Results and Discussion

### 3.1. Microstructural Analysis by SEM

Scanning electron microscopy (SEM) was employed to characterize the initial microstructures of Ti-CP2, Ti-6Al-2Sn-4Zr-2Mo, and Ti-6Al-4V. As shown in Figure 1a, Ti-CP2 exhibits a predominantly α-phase microstructure, consistent with the expected microstructural characteristics of commercially pure titanium. Ti-6Al-2Sn-4Zr-2Mo (Figure 1b) exhibits the characteristic heterogeneous morphology of a near-α titanium alloy, whereas Ti-6Al-4V (Figure 1c) shows a finer heterogeneous microstructure consistent with its α + β constitution. These observations reveal clear differences in microstructural morphology among the three titanium materials.

The corresponding EDS spectra are presented in Figure 1d–f. The spectrum obtained from Ti-CP2 (Figure 1d) was dominated by Ti, whereas Ti-6Al-4V (Figure 1f) showed Ti, Al, and V. For Ti-6Al-2Sn-4Zr-2Mo (Figure 1e), Ti, Al, Zr, Sn, and Mo were detected. The local semi-quantitative elemental compositions obtained from the analyzed areas are summarized in Table 2.

The microstructural differences observed among Ti-CP2, Ti-6Al-2Sn-4Zr-2Mo, and Ti-6Al-4V may influence their spontaneous electrochemical response by modifying the local characteristics of the alloy/electrolyte interface. Accordingly, these initial microstructural features are considered in the subsequent interpretation of the electrochemical noise response and its statistical and time–frequency analyses.

SEM examination also revealed microporosity, with pore sizes of approximately 0.5–1 µm, in the Ti-CP2 and Ti-6Al-4V samples. These features represent local microstructural heterogeneities that may influence the electrochemical response of the materials. However, their specific origin and direct contribution to the subsequent electrochemical activity were not experimentally established in the present study.

From an electrochemical perspective, these micropores may promote local variations in electrolyte accessibility and current distribution, potentially affecting the electrochemical behavior of the passive surface in the chloride-containing environment [46]. However, because the pre- and post-exposure SEM observations were not performed at identical surface locations, a direct spatial correlation between the initially observed micropores and subsequent electrochemical events cannot be established. Therefore, the observed microporosity is considered a possible source of local electrochemical heterogeneity rather than direct evidence of preferential sites for localized corrosion. Its possible influence is considered together with the EN time series, statistical parameters, and time–frequency analyses presented in the following sections [48,49].

### 3.2. Electrochemical Noise Analysis

#### 3.2.1. Time-Domain Analysis

Figure 2 and Figure 3 show the electrochemical potential noise (EPN) and electrochemical current noise (ECN) time series, respectively, for Ti-CP2, Ti-6Al-2Sn-4Zr-2Mo, and Ti-6Al-4V exposed to Ringer’s solution. Clear differences in the amplitude, frequency, and temporal distribution of the spontaneous electrochemical fluctuations can be observed among the three materials.

The EPN time series (Figure 2) shows that Ti-CP2 and Ti-6Al-2Sn-4Zr-2Mo exhibit comparatively smaller potential fluctuations during most of the exposure period, although isolated transients are observed. Ti-6Al-2Sn-4Zr-2Mo shows several discrete potential excursions, particularly during the intermediate and latter portions of the experiment, separated by relatively stable periods. In contrast, Ti-6Al-4V exhibits a considerably higher density of potential fluctuations and larger-amplitude transients throughout the experiment, indicating greater dynamic electrochemical activity at the alloy/electrolyte interface.

A similar distinction is observed in the ECN time series (Figure 3). Ti-CP2 and Ti-6Al-2Sn-4Zr-2Mo display relatively low-amplitude current fluctuations with occasional transient events, whereas Ti-6Al-4V exhibits substantially larger and more frequent current fluctuations over the entire acquisition period. The current transients of Ti-6Al-4V reach amplitudes on the order of 10^−3^ mA·cm^−2^, approximately one order of magnitude higher than those observed for Ti-6Al-2Sn-4Zr-2Mo. The higher density and amplitude of these current transients indicate more frequent spontaneous changes in the electrochemical state of the Ti-6Al-4V/electrolyte interface.

The transient behavior observed in both EPN and ECN is consistent with a dynamically evolving passive interface. Relatively stable portions of the signals can be associated with periods of lower electrochemical activity, whereas abrupt potential and current excursions indicate rapid changes at the metal/electrolyte interface. In passive titanium alloys, such fluctuations may arise from local perturbations of the oxide film followed by its recovery. However, the EN time series alone cannot unambiguously distinguish passive-film breakdown/repassivation from other interfacial processes or establish the occurrence of stable localized corrosion. Therefore, these observations were subsequently evaluated together with the statistical parameters, EMD, Hilbert time–frequency analysis, and surface characterization.

The statistical parameters obtained from the EN signals are summarized in Table 3. Skewness and kurtosis were used to compare the distribution and transient characteristics of the electrochemical fluctuations among the three titanium materials, rather than as direct indicators of specific corrosion mechanisms [31,32,35,50].

Table 3 summarizes the statistical parameters obtained from the electrochemical noise (EN) measurements for Ti-CP2, Ti-6Al-2Sn-4Zr-2Mo, and Ti-6Al-4V exposed to Ringer’s solution. Significant differences in noise resistance (Rn) were observed among the investigated materials. Ti-6Al-2Sn-4Zr-2Mo exhibited the highest Rn value (45,412 ± 57 Ω·cm^2^), followed by Ti-6Al-4V (11,784 ± 39 Ω·cm^2^) and Ti-CP2 (4114 ± 45 Ω·cm^2^), indicating greater resistance to electrochemical fluctuations for the Zr- and Mo-containing alloy. Relatively low localization index (LI) values were obtained for all investigated materials, with Ti-CP2 exhibiting 0.02634 ± 0.002, Ti-6Al-2Sn-4Zr-2Mo 0.04 ± 0.002, and Ti-6Al-4V 0.06 ± 0.002. Kurtosis values higher than 3 were obtained for all materials, indicating non-Gaussian distributions characterized by intermittent electrochemical transients. Negative skewness values were also observed, with Ti-CP2 (−0.6 ± 0.06) and Ti-6Al-2Sn-4Zr-2Mo (−0.5 ± 0.06) exhibiting greater signal asymmetry than Ti-6Al-4V (−0.1 ± 0.04). These statistical parameters demonstrate distinct electrochemical fluctuation behavior among the three titanium materials. However, identification of the predominant electrochemical processes should not rely exclusively on individual statistical parameters; therefore, these results are further correlated with the EMD and Hilbert time–frequency analyses presented in the following section.

#### 3.2.2. Time–Frequency Domain Analysis

Figure 4 shows the empirical mode decomposition (EMD) of the electrochemical noise signals obtained for Ti-CP2, Ti-6Al-2Sn-4Zr-2Mo, and Ti-6Al-4V in Ringer’s solution. The decomposition revealed differences in the number and characteristics of the intrinsic mode functions (IMFs) among the investigated materials. The Ti-CP2 signal was decomposed into four IMFs, whereas Ti-6Al-2Sn-4Zr-2Mo exhibited five IMFs and Ti-6Al-4V six IMFs, in addition to the corresponding residual component.

The larger number of IMFs observed for Ti-6Al-4V indicates that its electrochemical noise signal contains fluctuations distributed over a broader range of characteristic time scales. In addition, the first IMFs exhibit numerous fluctuations and transient events, indicating a broader distribution of the higher-frequency components of the electrochemical response. In contrast, Ti-CP2 exhibited fewer intrinsic modes, with more clearly defined transient events concentrated in the first IMFs. Ti-6Al-2Sn-4Zr-2Mo showed an intermediate decomposition pattern, with transient activity distributed mainly among the higher-frequency IMFs.

At the lower-frequency modes, the signals progressively evolve toward smoother oscillatory components, while the residual represents the long-term trend of the original electrochemical noise signal. These differences in the EMD response demonstrate that the three titanium materials exhibit distinct temporal characteristics of electrochemical activity in Ringer’s solution [51].

Figure 5 shows the time–frequency–amplitude Hilbert spectra obtained from the electrochemical current noise (ECN) signals of Ti-CP2, Ti-6Al-2Sn-4Zr-2Mo, and Ti-6Al-4V during exposure to Ringer’s solution. The HHT analysis reveals differences in the temporal evolution and frequency distribution of the spontaneous electrochemical fluctuations among the three materials. In general, In general, contributions at relatively low frequencies may be associated with slower interfacial processes, including ionic transport and passive-film evolution, whereas short-duration components extending toward intermediate and higher frequencies may reflect faster electrochemical events related to local perturbation and subsequent recovery of the passive film.

For Ti-CP2 (Figure 5a), relatively low electrochemical activity is observed during the initial and intermediate stages of the experiment. The most significant fluctuations are concentrated mainly after approximately 2000–2500 s, where discrete transients of higher relative amplitude become evident. Although the response is dominated by low-frequency components, the occurrence of short-duration transients extending toward intermediate frequencies indicates occasional rapid perturbations of the surface film. The absence of sustained high-amplitude activity suggests that these events are transient rather than continuously propagating localized corrosion processes.

Ti-6Al-2Sn-4Zr-2Mo (Figure 5b) exhibits a different temporal distribution, with relatively low-amplitude fluctuations during the intermediate stage and more pronounced transient activity at approximately 2500–3400 s. Contributions occur predominantly at low frequencies, accompanied by discrete components extending toward intermediate frequencies. This behavior suggests the coexistence of slower passive-film-related processes and intermittent faster electrochemical events. The transient nature of these events is consistent with localized destabilization followed by recovery of the passive interface, rather than sustained electrochemical activity.

The Hilbert spectrum of Ti-6Al-4V (Figure 5c) exhibits the broadest temporal and frequency distribution of electrochemical fluctuations. Numerous low-amplitude events occur over a substantial fraction of the exposure period, together with more intense transient events during the intermediate and final stages. This broader time–frequency distribution indicates a more dynamically heterogeneous electrochemical response. The coexistence of low-frequency activity with faster transient components suggests repeated perturbation–recovery events at the passive interface rather than a temporally uniform passive condition.

Overall, the Hilbert spectra reveal distinct temporal and frequency characteristics for the three titanium materials. Ti-CP2 exhibits electrochemical activity concentrated mainly at longer exposure times, Ti-6Al-2Sn-4Zr-2Mo shows intermittent activity while maintaining a comparatively high resistance to electrochemical fluctuations, whereas Ti-6Al-4V displays the broadest and most persistent distribution of transient activity. Importantly, the Hilbert spectra do not by themselves demonstrate stable pitting; rather, the intermittent character of the signals is more consistent with transient passive-film destabilization and subsequent recovery of the passive interface. This interpretation is consistent with the statistical EN parameters and should be considered together with the SEM/EDS results to establish the predominant electrochemical behavior of the investigated materials [52,53,54,55].

### 3.3. SEM/EDS Surface Analysis

Following the electrochemical tests, the surfaces of the three titanium materials were examined by scanning electron microscopy coupled with energy-dispersive X-ray spectroscopy (SEM/EDS) to evaluate their surface morphology and local elemental composition. Figure 6, Figure 7 and Figure 8 show the SEM-BSE micrographs and corresponding EDS spectra of Ti-CP2, Ti-6Al-2Sn-4Zr-2Mo, and Ti-6Al-4V, respectively, after electrochemical exposure to Ringer’s solution. The elemental compositions obtained from the selected regions are summarized in Table 4.

For Ti-CP2 (Figure 6), the analyzed region was predominantly composed of Ti (97.64 wt.%), with minor amounts of Si (1.88 wt.%) and Cl (0.48 wt.%). Oxygen was not detected in the analyzed region. The SEM-BSE micrograph (Figure 6a) shows a relatively uniform surface morphology, where the parallel linear features correspond to grinding and polishing marks from specimen preparation. No evident severe localized attack is observed within the analyzed area. The low Cl content indicates a limited presence of electrolyte-derived species on the surface after electrochemical exposure. However, because EDS probes (Figure 6b) a relatively large interaction volume, the absence of detectable oxygen should not be interpreted as evidence for the absence of the naturally formed passive oxide film.

For Ti-6Al-2Sn-4Zr-2Mo (Figure 7a), the SEM-BSE micrograph shows residual grinding and polishing marks, with no evident severe localized attack within the examined area. The main alloying elements Ti, Al, Zr, Mo, and Sn were detected in both analyzed regions. Region 1 (Figure 7b)was predominantly composed of Ti (79.33 wt.%), together with Al (7.28 wt.%), Zr (4.69 wt.%), Mo (2.40 wt.%), Sn (1.70 wt.%), and O (3.81 wt.%). In contrast, Region 2 (Figure 7c) exhibited a substantially higher O content (32.59 wt.%) and a lower Ti content (48.87 wt.%), while Al, Zr, Mo, and Sn remained detectable. Minor Si contents of 0.80 and 1.01 wt.% were also detected in Regions 1 and 2, respectively. Because the specimens were ground with SiC abrasive paper before electrochemical testing, the detected Si may be associated with residual surface contamination from specimen preparation; however, its origin cannot be conclusively established from EDS alone. These differences indicate marked local compositional heterogeneity on the alloy surface after electrochemical exposure. The higher oxygen content detected in Region 2 indicates greater local accumulation of oxygen-containing species; however, because of the EDS interaction volume, this observation should not be interpreted as a direct measurement of passive-film composition or thickness.

For Ti-6Al-4V (Figure 8a), the SEM-BSE micrograph shows residual grinding and polishing marks, with no evident severe localized attack within the examined area. The principal alloying elements Ti, Al, and V were detected in both analyzed regions. Region 1 (Figure 8b) was predominantly composed of Ti (81.01 wt.%), together with Al (7.11 wt.%), V (2.48 wt.%), O (8.89 wt.%), and a minor Si content (0.51 wt.%). In contrast, Region 2 (Figure 8c) exhibited a higher O content (14.20 wt.%) and a lower Ti content (70.35 wt.%), while Al (6.07 wt.%) and V (2.45 wt.%) remained at comparable levels. Cl was also detected in Region 2 (6.94 wt.%), indicating a localized presence of chlorine-containing species within the analyzed surface region after electrochemical exposure. The compositional differences between the two regions indicate local surface heterogeneity following exposure to Ringer’s solution.

Overall, the SEM/EDS results reveal local morphological and compositional heterogeneity on the surfaces of the investigated titanium materials after electrochemical exposure. Nevertheless, these results should be interpreted cautiously. The interaction volume associated with EDS is considerably larger than the thickness of the naturally formed passive film; therefore, variations in oxygen content cannot be considered direct evidence of the composition, continuity, thickness, or protective character of the passive oxide layer. Similarly, the detection of Cl in a selected region of Ti-6Al-4V indicates the local presence of chlorine-containing species within or near the analyzed surface region but does not, by itself, demonstrate chloride incorporation or penetration into the passive film [22,56,57,58,59].

Accordingly, the SEM/EDS observations are interpreted as complementary evidence of local surface changes and compositional heterogeneity following electrochemical exposure rather than as direct characterization of passive-film chemistry or integrity. When considered together with the statistical EN, EMD, and Hilbert time–frequency analyses, these observations support the existence of distinct electrochemical responses among Ti-CP2, Ti-6Al-2Sn-4Zr-2Mo, and Ti-6Al-4V in Ringer’s solution.

The combined electrochemical noise and surface characterization results reveal distinct electrochemical responses among the investigated titanium materials in Ringer’s solution. The statistical EN parameters showed clear differences in noise resistance and transient characteristics, while EMD and Hilbert time–frequency analyses demonstrated that the electrochemical fluctuations were distributed differently in time and frequency for each material. The ability of Hilbert–Huang-based analysis to resolve electrochemical noise transients simultaneously in the time and frequency domains and to distinguish different patterns of electrochemical activity has been demonstrated in previous corrosion studies [30,60]. Ti-6Al-2Sn-4Zr-2Mo exhibited the highest noise resistance and intermittent transient activity, whereas Ti-6Al-4V showed a broader and more persistent distribution of electrochemical fluctuations. Ti-CP2 exhibited the lowest noise resistance and comparatively high kurtosis, consistent with a greater contribution of discrete transient events. These differences are complemented by the SEM/EDS observations, which revealed localized compositional heterogeneity and different degrees of accumulation of electrolyte-derived species on the exposed surfaces. Taken together, the results indicate that the three titanium materials exhibit distinct passive electrochemical responses, characterized by different degrees of interfacial fluctuation and transient surface activity rather than unequivocal evidence of stable localized corrosion [30,61].

Although the combination of statistical electrochemical noise analysis, EMD, Hilbert time–frequency analysis, and SEM/EDS provides complementary information on the electrochemical behavior of the investigated titanium materials, these techniques do not directly determine the electrochemical properties or chemical structure of the passive films. Previous investigations of titanium and titanium alloys in physiological environments have demonstrated that EIS can provide information on the electrical and interfacial properties of passive films, whereas surface-sensitive techniques such as XPS can identify their chemical composition and oxidation states [61]. Studies performed in Ringer’s and simulated physiological solutions have also shown that combining electrochemical measurements with surface characterization provides a more complete description of the structure and protective behavior of passive films on titanium alloys [23,62,63,64]. Future studies combining these approaches could therefore contribute to a more detailed understanding of passive-film stability and degradation processes in titanium materials exposed to Ringer’s solution and related simulated physiological environments.

## 4. Conclusions

1. Statistical electrochemical noise analysis revealed distinct electrochemical responses among Ti-CP2, Ti-6Al-2Sn-4Zr-2Mo, and Ti-6Al-4V in Ringer’s solution. Ti-6Al-2Sn-4Zr-2Mo exhibited the highest noise resistance (45,412 ± 57 Ω·cm^2^), followed by Ti-6Al-4V (11,784 ± 39 Ω·cm^2^) and Ti-CP2 (4114 ± 45 Ω·cm^2^), indicating greater resistance to electrochemical fluctuations for Ti-6Al-2Sn-4Zr-2Mo under the investigated conditions.
2. Relatively low localization index values were obtained for Ti-CP2 (0.02634 ± 0.002), Ti-6Al-2Sn-4Zr-2Mo (0.04 ± 0.002), and Ti-6Al-4V (0.06 ± 0.002). Kurtosis values greater than 3 and negative skewness values revealed non-Gaussian ECN distributions characterized by different degrees of asymmetry and contributions from intermittent transient events. These statistical parameters further differentiate the electrochemical responses of the three titanium materials.
3. EMD and Hilbert time–frequency–amplitude analyses revealed differences in the temporal and frequency characteristics of the ECN signals. Ti-CP2, Ti-6Al-2Sn-4Zr-2Mo, and Ti-6Al-4V exhibited four, five, and six IMFs, respectively, while Ti-6Al-4V showed the broadest and most persistent distribution of transient activity. The intermittent nature of the electrochemical fluctuations is consistent with dynamic passive-interface processes involving transient perturbation and recovery rather than providing unequivocal evidence of stable localized corrosion.
4. SEM/EDS observations revealed local post-exposure morphological and compositional heterogeneity among the investigated titanium materials. Variations in oxygen content and the localized detection of chlorine-containing species were observed in selected regions; however, these results were interpreted as complementary evidence of surface changes rather than as direct characterization of passive-film composition, thickness, or integrity.
5. The significance of this study lies in the integrated application of statistical EN parameters, EMD, Hilbert time–frequency–amplitude analysis, and SEM/EDS to comparatively evaluate Ti-CP2, Ti-6Al-2Sn-4Zr-2Mo, and Ti-6Al-4V under identical exposure conditions in Ringer’s solution. This complementary approach enables differences in electrochemical fluctuation resistance, transient dynamics, and post-exposure surface characteristics to be distinguished without relying on a single electrochemical parameter or assigning corrosion mechanisms solely from statistical criteria. The results therefore provide a more comprehensive framework for evaluating the dynamic passive behavior of titanium materials under the investigated Ringer’s solution conditions.

## Figures and Tables

**Figure 1:**
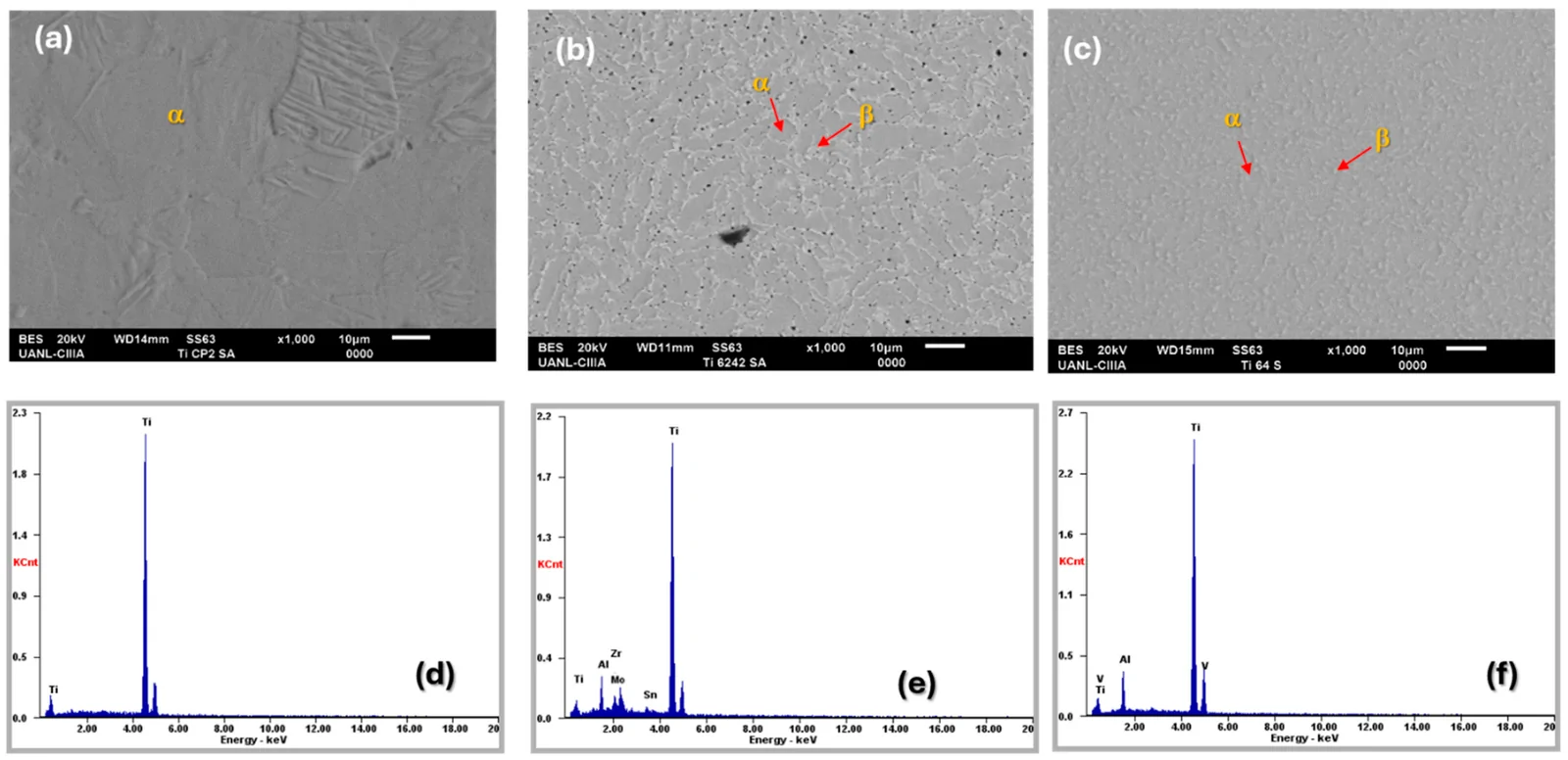
SEM-BSE micrographs and corresponding EDS spectra of the titanium materials in the initial condition: (**a**) Ti-CP2; (**b**) Ti-6Al-2Sn-4Zr-2Mo; (**c**) Ti-6Al-4V; and corresponding EDS spectra of (**d**) Ti-CP2, (**e**) Ti-6Al-2Sn-4Zr-2Mo, and (**f**) Ti-6Al-4V. The α and β microstructural constituents are indicated where applicable.

**Figure 2:**
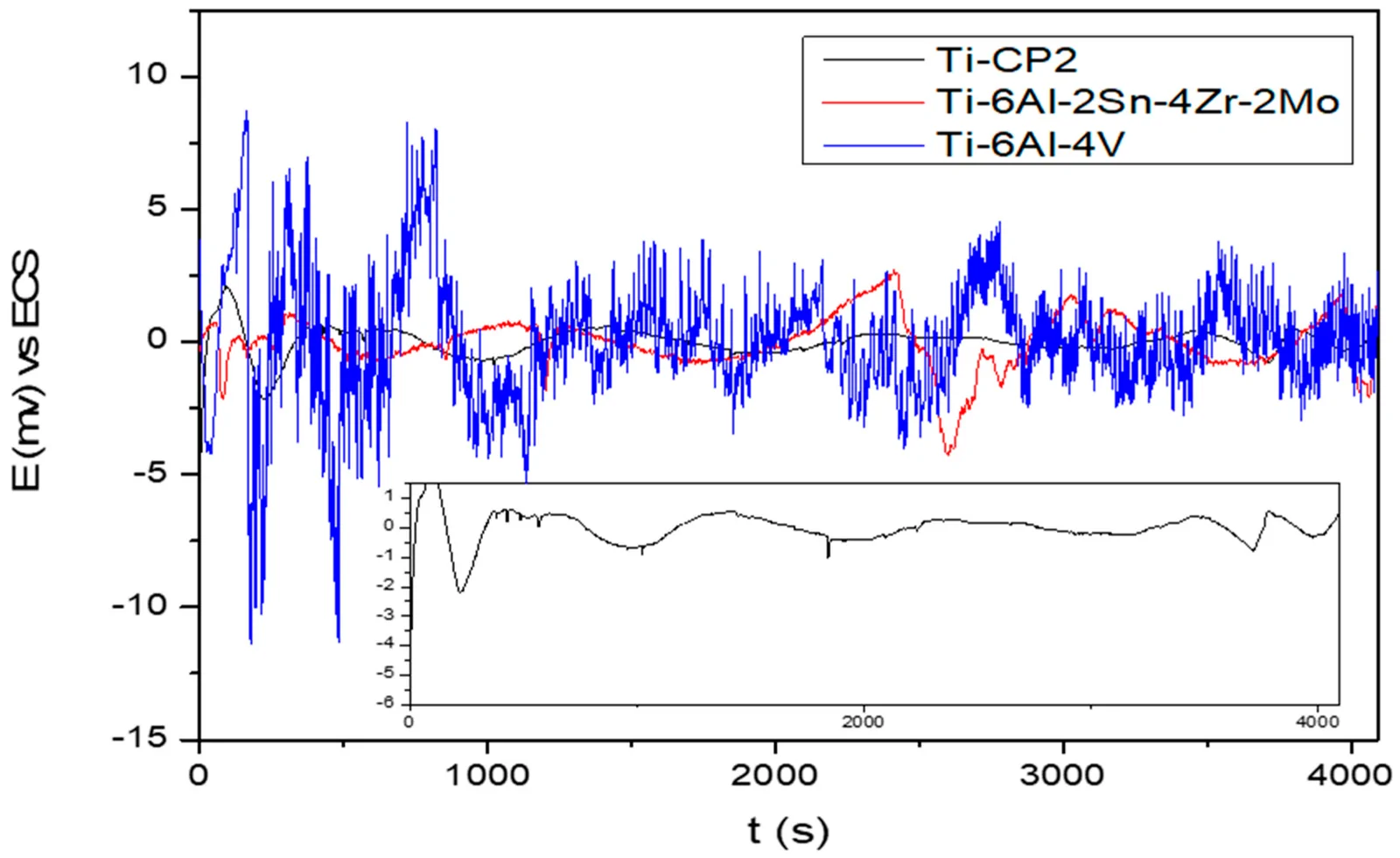
EPN time series of Ti alloy exposure to Ringer’s solution.

**Figure 3:**
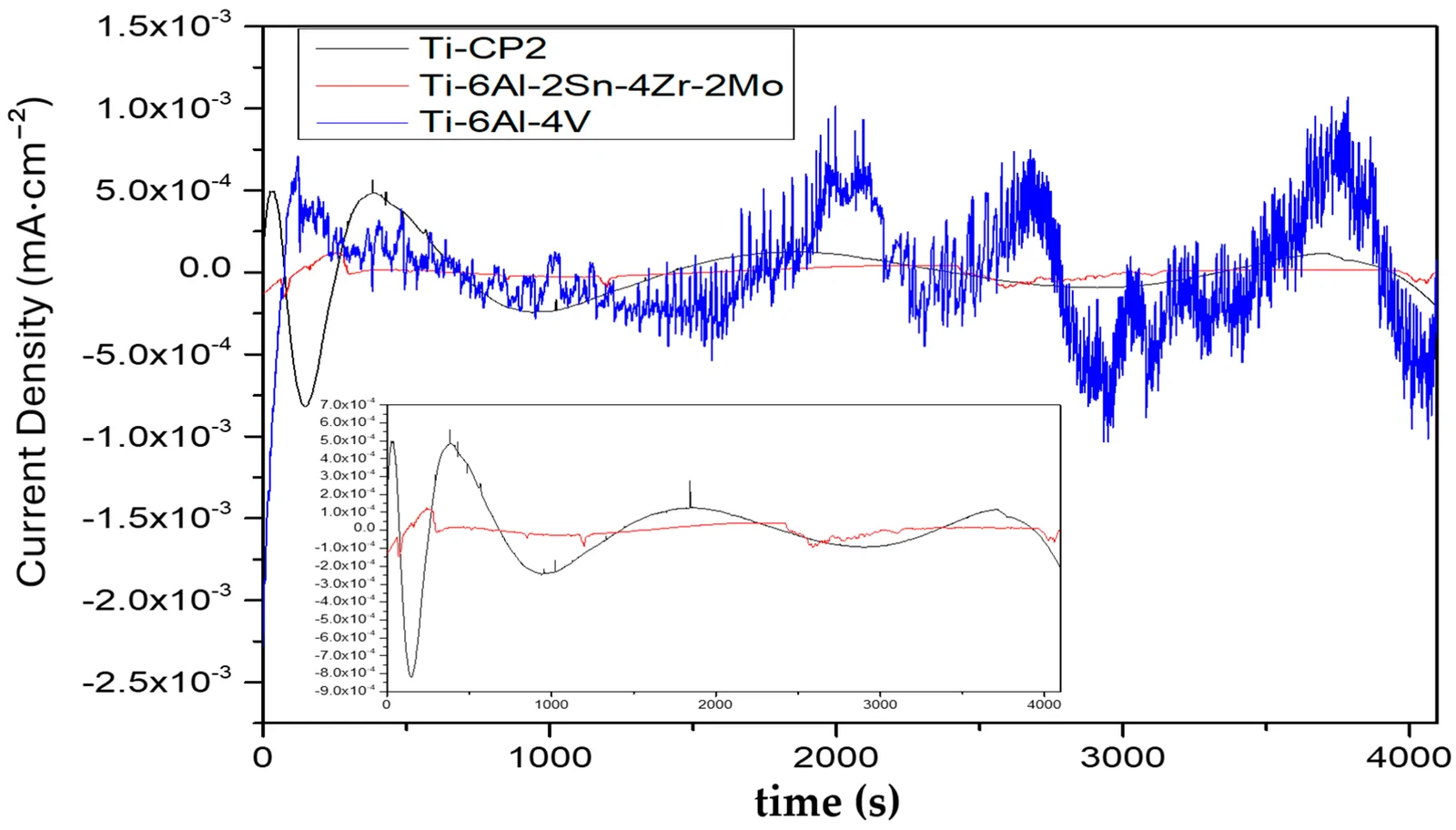
ECN time series of Ti alloy exposure to Ringer’s solution.

**Figure 4:**
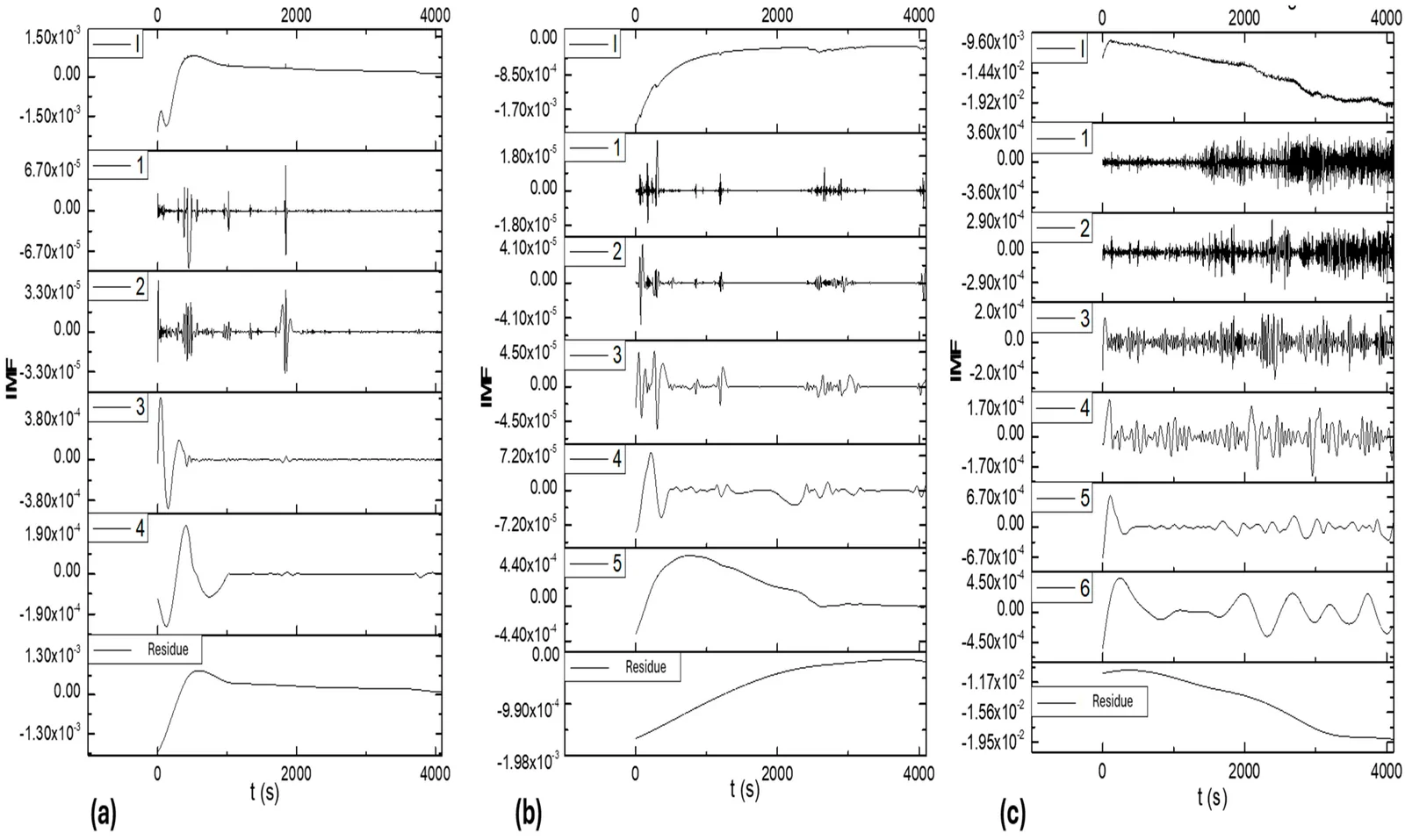
Empirical mode decomposition (EMD) of the electrochemical current noise (ECN) signals for (**a**) Ti-CP2, (**b**) Ti-6Al-2Sn-4Zr-2Mo, and (**c**) Ti-6Al-4V exposed to Ringer’s solution.

**Figure 5:**
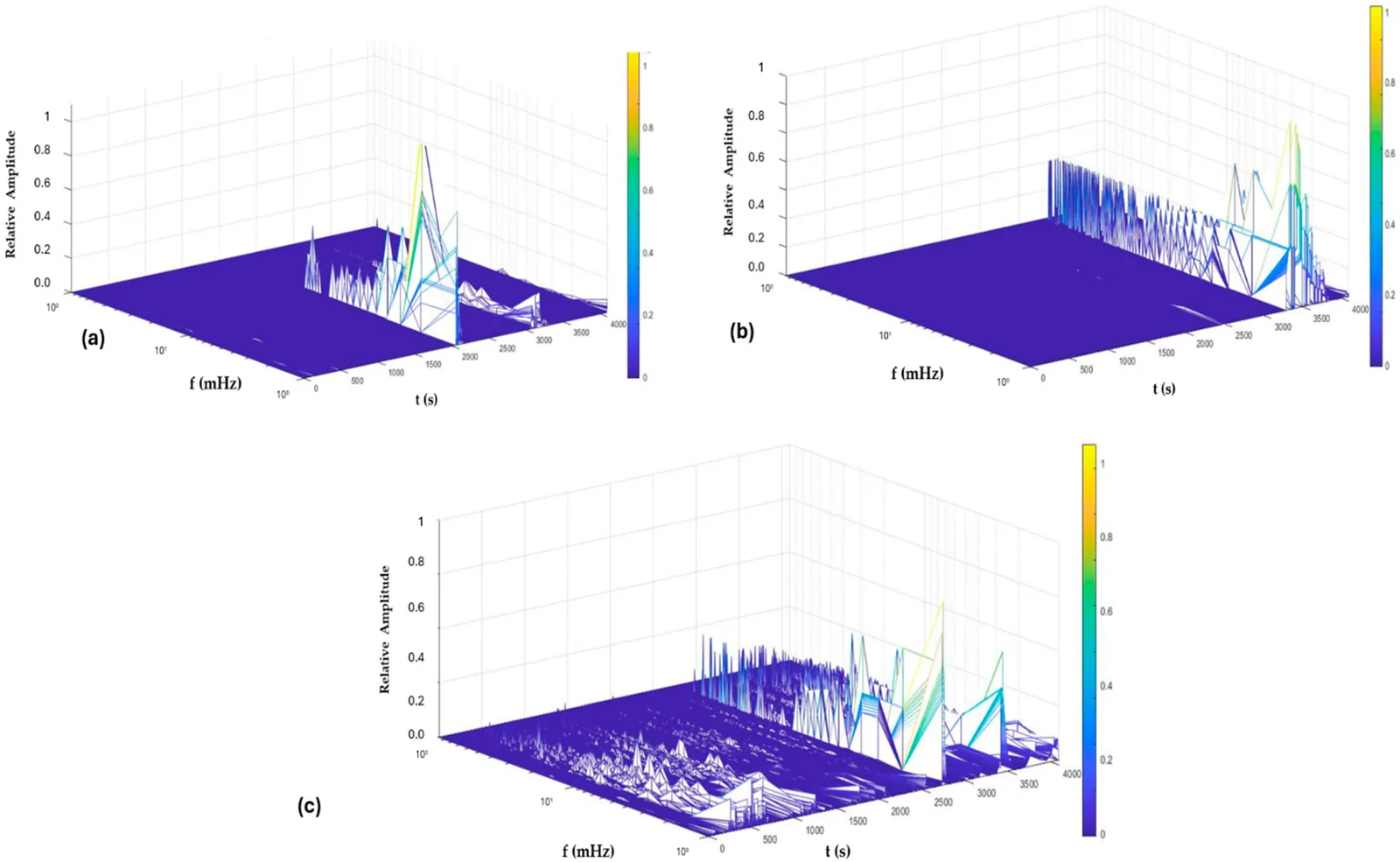
Hilbert time–frequency–amplitude spectra obtained from the electrochemical current noise (ECN) signals of the titanium materials exposed to Ringer’s solution: (**a**) Ti-CP2, (**b**) Ti-6Al-2Sn-4Zr-2Mo, and (**c**) Ti-6Al-4V.

**Figure 6:**
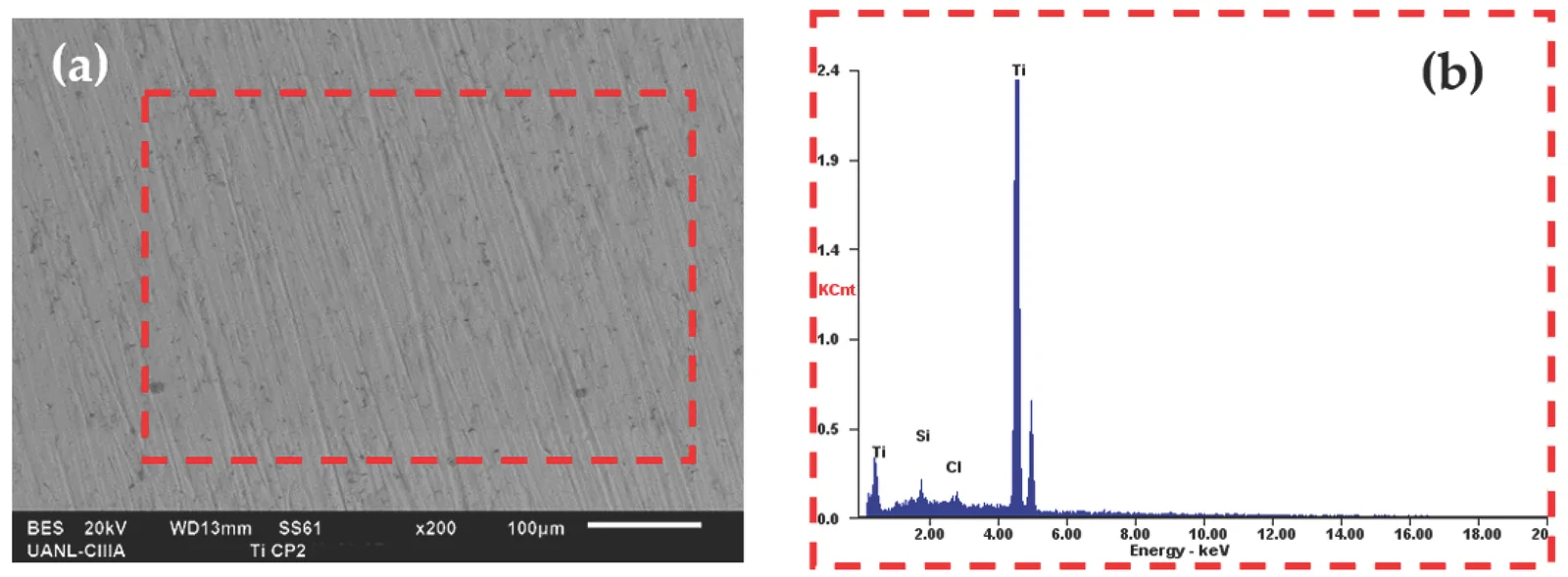
SEM-BSE surface morphology and EDS analysis of Ti-CP2 after electrochemical exposure to Ringer’s solution: (**a**) SEM-BSE micrograph showing Region 1 (red rectangle) and (**b**) EDS spectrum of Region 1.

**Figure 7:**
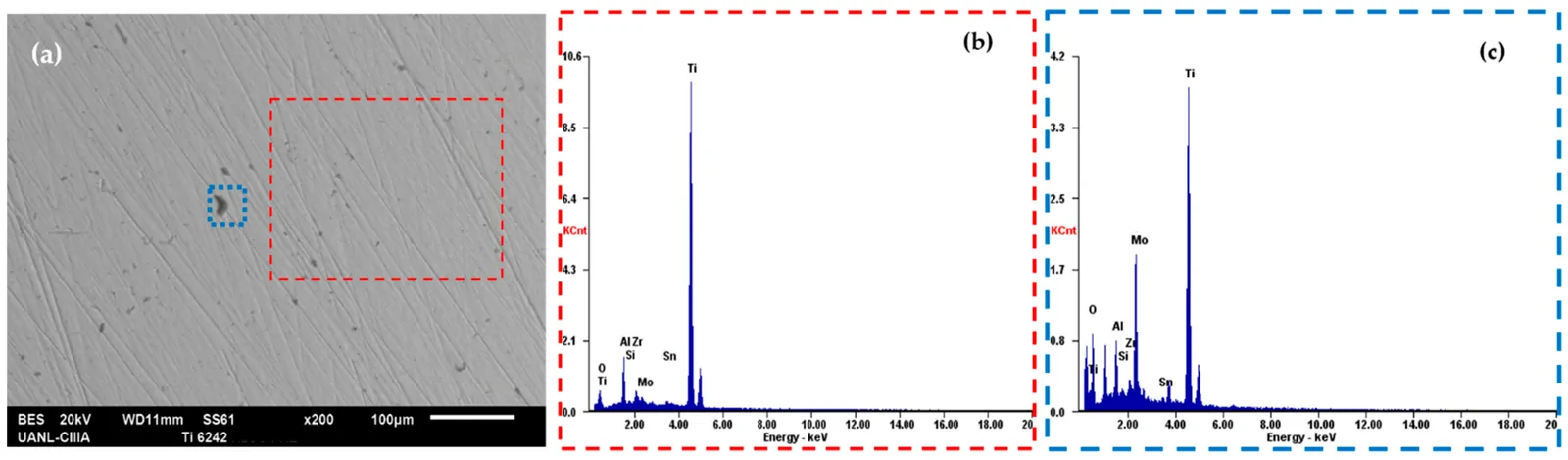
SEM-BSE surface morphology and EDS analysis of Ti-6Al-2Sn-4Zr-2Mo after electrochemical exposure to Ringer’s solution: (**a**) SEM-BSE micrograph showing Region 1 (red rectangle) and Region 2 (blue rectangle); (**b**) EDS spectrum of Region 1; and (**c**) EDS spectrum of Region 2.

**Figure 8:**
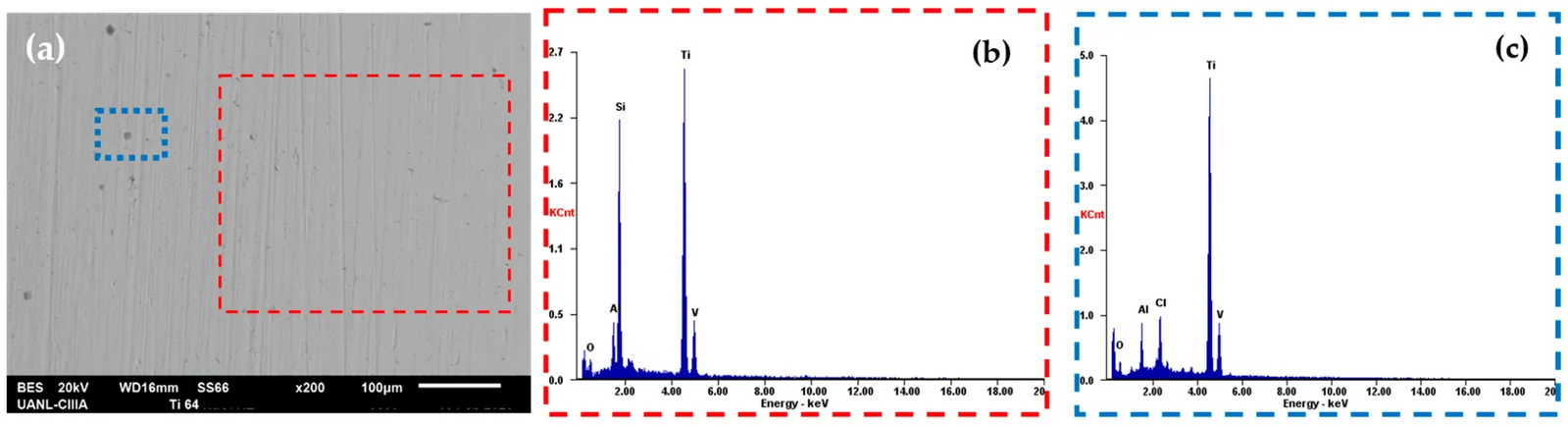
Post-exposure SEM-BSE surface morphology and EDS analysis of Ti-6Al-4V after electrochemical testing in Ringer’s solution: (**a**) SEM-BSE micrograph showing Region 1 (red rectangle) and Region 2 (blue rectangle); (**b**) EDS spectrum of Region 1; and (**c**) EDS spectrum of Region 2.

**Table 1:** Chemical composition (wt.%) of the titanium materials investigated in this study.

Titanium Alloy	Ti	Al	V	Zr	Mo	Sn	Fe
Ti-CP2	99.94 ± 0.005	---	---	---	---	---	0.038 ± 0.005
Ti-6Al-2Sn-4Zr-2Mo	84.65 ± 0.19	6.75 ± 0.20	---	4.18 ± 0.01	1.99 ± 0.008	2.08 ± 0.01	---
Ti-6Al-4V	87.71 ± 0.36	7.14 ± 0.37	4.03 ± 0.08	---	---	---	0.21 ± 0.01

**Table 2:** Local semi-quantitative elemental composition (wt.%) obtained by EDS from the analyzed areas of the titanium materials in the initial condition.

Material	Ti	Al	V	Zr	Sn	Mo
Ti CP2	100.00	—	—	—	—	
Ti-6Al-2Sn-4Zr-2Mo	85.56	6.63	—	4.16	1.98	1.67
Ti-6Al-4V	91.52	7.66	0.82	—	—	

**Table 3:** Statistical electrochemical noise parameters obtained for the titanium materials exposed to Ringer’s solution.

Material	Electrolyte	R_n_ (Ω·cm^2^)	LI	Kurtosis	Skewness
Ti-CP2	Ringer’s	4114 ± 45	0.02634 ± 0.002	8 ± 0.09	−0.6 ± 0.06
Ti-6Al-2Sn-4Zr-2Mo	Ringer’s	45,412 ± 57	0.04 ± 0.002	6 ± 0.09	−0.5 ± 0.06
Ti-6Al-4V	Ringer’s	11,784 ± 39	0.06 ± 0.002	5 ± 0.08	−0.1 ± 0.04

**Table 4:** Local semi-quantitative elemental composition (wt.%) obtained by EDS from selected regions of the titanium materials after electrochemical exposure to Ringer’s solution.

Material	EDS Region	O	Al	Si	Cl	Ti	V	Zr	Mo	Sn
Ti-CP2	Region 1 (Red)	—	—	1.88	0.48	97.64	—	—	—	—
Ti-6Al-2Sn-4Zr-2Mo	Region 1 (Red)	3.81	7.28	0.80	—	79.33	—	4.69	2.40	1.70
Ti-6Al-2Sn-4Zr-2Mo	Region 2 (Blue)	32.59	6.23	1.01	—	48.87	—	3.77	5.93	1.60
Ti-6Al-4V	Region 1 (Red)	8.89	7.11	0.51	—	81.01	2.48	—	—	—
Ti-6Al-4V	Region 2 (Blue)	14.20	6.07	—	6.94	70.35	2.45	—	—	—

Note: “—“not detected.

## Data Availability

The original contributions presented in this study are included in the article. Further inquiries can be directed to the corresponding authors.
